# Biodegradable synthetic polymers for biomedical and tissue engineering applications: tailoring degradation kinetics with tissue regeneration timeline

**DOI:** 10.1186/s12938-026-01550-5

**Published:** 2026-05-05

**Authors:** Syafiqah Saidin, Saiful Irwan Zubairi, Jaweria Ambreen, Nurul Asmak Md Lazim, Madeeha Sadia, Jocelyn Tze Wei Lim, Mokhamad Fakhrul Ulum, Aisyah Elliyanti, Farshid Sefat

**Affiliations:** 1 IJN-UTM Cardiovascular Engineering Centre, Institute of Human Centered Engineering, Universiti Teknologi Malaysia, UTM, 81310 Johor Bahru, Johor Malaysia; 2 Department of Biomedical Engineering & Health Sciences, Faculty of Electrical Engineering, Universiti Teknologi Malaysia, UTM, 81310 Johor Bahru, Johor Malaysia; 3 Department of Food Sciences, Faculty of Science & Technology, Universiti Kebangsaan Malaysia, UKM, 43600 Bangi, Selangor Malaysia; 4 Department of Chemistry, COMSATS University Islamabad, Park Road, Islamabad, 45550 Pakistan; 5 Department of Bioprocess Engineering and Polymer, School of Chemical & Energy Engineering, Faculty of Engineering, Universiti Teknologi Malaysia, UTM, 81310 Johor Bahru, Johor Malaysia; 6 Department of Biomedical Engineering, NED University of Engineering & Technology, Karachi, Pakistan; 7 School of Biomedical Sciences, Faculty of Medical Sciences, Newcastle University, Newcastle Upon Tyne, UK; 8 Division of Reproduction, Obstetrics and Gynaecology, School of Veterinary Medicine and Biomedical Sciences, IPB University, Bogor, Indonesia; 9 Faculty of Medicine, Andalas University, Padang City, West Sumatra 25175 Indonesia; 10 Biomedical Engineering Discipline, Faculty of Engineering and Digital Technologies, University of Bradford, Bradford, BD7 1DP UK

**Keywords:** Biodegradable, Synthetic polymer, Biomedical, Tissue engineering, Degradation kinetics

## Abstract

The adoption of biodegradable synthetic polymers in biomedical and tissue engineering becomes a focal point, offering alternative solutions to organ transplantation and conventional permanent restoration. The key principle in developing scaffold-based for physiological implantation is the synchronisation of polymer’s degradation kinetics with the regeneration timeline of host tissues. Different implantation lesions exhibit vastly different healing durations, ranging from a few weeks to several months or years. A mismatch timeline can be detrimental where premature degradation will remove the physical framework needed for cell integration, whereas overly slow degradation will restrict spaces for new tissue growth. This review study provides a comprehensive discussion on the degradation mechanisms of biodegradable synthetic polymers in physiological environments. Four widely studied polymers—polylactic acid (PLA), polyvinyl alcohol (PVA), polycaprolactone (PCL), and polyurethane (PU)—were reviewed in depth on the degradation mechanisms and influencing factors. Scientific experimental data from the previous studies were summarised, including degradation percentage, experimental conditions, degradation timeline, and estimated complete degradation period. Specifically, four degradation mechanisms are associated with the degradation of synthetic polymers in physiological environments including chemical hydrolysis, enzymatic-mediated metabolism, oxidative degradation, and pH-dependent degradation. Each of the mechanisms may act independently or synergistically under different biological conditions. The degradation of polymers is accordingly influenced by the chemical structures, fabrication routes, degradation pathways, and physicochemical factors. These data are correlated with their optimal use in biomedical and TE applications for fast regenerating tissues to slow-healing or load-bearing structures. Comprehensively, PVA is aligned well with short-to-intermediate healing tissues such as the skin and the cornea due to its high degradation capability, while PLA, PCL, and PU that degrade from weeks to years are suitable for mediate-healing soft tissues to long-term implantations such as load-bearing bone, cartilage, ligament, neural, and vascular implantations. By aligning polymer degradation profiles with the biological timelines of tissue regeneration, this review provides a translational framework for synthetic polymer selection to enable optimum scaffold’s functionalities and clinical outcomes.

## Introduction

Limited availability of donated organs and timeline accessibility, along with post-transplant complications, have increased the mortality rate among patients with chronic diseases [1–3]. Although conventional approaches (e.g., the implantation of synthetic prostheses/substitutes to replace failed organs and the utilisation of mechanical devices to assist organ functionalities) have improved patients’ condition, the patients require toleration with subsequence procedures to resume their normal daily life [4–6]. Furthermore, these procedures are accompanied by common post-operative issues such as tissue toxicity, immune rejection, inflammation, and bacterial infection [7–9]. To alleviate these limitations, tissue engineering (TE) in combination with biomaterials is known to be the most reliable alternative to replace the procedure of organ transplantation in supporting successful tissue regeneration [10, 11]. The progress in TE has evolved through the exploration and development of polymeric biodegradable materials that are defined as a three-dimensional (3D) framework capable of being used as a template to induce cell growth and support tissue regeneration while averting inflammation and restoring the normal operation of damaged tissues and organs [12–14].

Polymeric biodegradable materials can be classified into natural and synthetic polymers [15, 16]. Natural-based polymers generally exhibit physicochemical and biological properties analogous to the physiological tissues. Despite the biofunctional and bioactivity of natural-based polymers, synthetic polymers are worth expanding research, owing to their stability, mechanical integrity, uniform framework, sustainable degradation, versatile processing modification, and feasible resources [17]. In addressing the principle of TE, those temporary polymer frameworks should be biodegradable for cell penetration and tissue integration. Biodegradable scaffolds possess an ability to degrade simultaneously in the surrounding aqueous environment without the need for surgical removal [18]. The degraded area will anchor cell attachment and tissue integration, mediating the framework of extracellular matrix (ECM) for tissue regeneration and organ reconstruction [19]. Biodegradable scaffolds which imitate physiological ECM of indigenous tissues can be loaded with specific biofunctional materials and biomolecules to carry the action of drug delivery carrier in supporting multiple domain regenerations (e.g., the skin, vascular, bone, cornea, and nervous) [20–22].

The desired scaffold materials must exhibit excellent features of biocompatibility, biofunctionality, tolerable biodegradation, adequate strength, and extremely low immunological reaction [23]. Biocompatibility involves the favourable interaction of biomaterials with the cellular environment without or with minimum complications, such as inflammation, toxicity, mutagenicity, carcinogenicity, or antigenicity [24]. In this regard, the use of synthetic biodegradable polymers offers a facile biocompatibility tuning while providing adequate blood–material interaction and mechanical strength to accommodate successful clinical settings [25]. The compliance between the polymeric scaffold and adjacent cells is significantly influenced by the intrinsic properties of the scaffold’s material [26]. As an example, for the normalisation of cell functions such as their activation, proliferation, and adhesion; biocompatibility as well as in-match biochemistry between cells and the scaffold, is crucial. Moreover, various scaffold characteristics influence the kinetics of degradation and the effectiveness of tissue regeneration. These include chemical properties (e.g., composition and surface functionality), structural features (e.g., morphology, porosity, and pore size distribution), and physical characteristics (e.g., crystallinity and wettability) [27]. Therefore, a primary comprehension of the mechanism of polymer degradation is mandatory in designing and developing polymeric scaffolds for TE settings. 

Another important trait to be emphasised in polymer selection is the biodegradable timeline where different types of tissue require different timelines for complete remodelling/regeneration in the human body. Generally, some tissues are regenerated in a few weeks and others may reach more than a year for complete regeneration, depending upon the age and health history of an individual [28, 29]. Table 1 presents the common regeneration timelines for various types of tissue in the human body. Biological factors that affect tissue regeneration are also listed, which mainly involve growth factor expression, inflammatory and immune responses, cell proliferation, vascularisation, ECM remodelling and reconstruction, and loading of physiological forces.

**Table 1:** Regeneration timelines and biological factors affecting tissue regeneration for various types of tissues in the human body [10, 28, 29]

Tissue	Regeneration timeline	Biological factors affecting tissue regeneration
Skin	3 days to ≥1 year	• Expression of growth factors such as EGF, FGF, VEGF, TGF-β, etc. • Inflammatory cytokines and immune responses • Migration and proliferation of keratinocytes and fibroblasts • Adequate vascularisation and oxygen supply for angiogenesis • ECM remodelling and collagen deposition • Loading of physiological forces such as wound contraction • Stem cell activities from hair follicles and basal layer
Bone	6 to ≥12 weeks	• Expression of bone morphogenetic proteins such as BMP-2 and BMP-7 • Inflammatory cytokines and immune responses • Osteoblast, osteoclast, and osteocyte activities • Availability of bone minerals composition such as calcium, phosphate, and vitamin D • Adequate vascularisation and oxygen supply for angiogenesis and osteogenesis • Loading of physiological forces such as dynamic and static loading, in pair with mechanical stability
Cartilage	6 weeks to ≥24 months	• Expression of growth factors such as TGF-β, IGF-1, FGF, etc. • Capacity of chondrocyte proliferation • Presence of MSCs • ECM composition such as type II collagen and proteoglycans • Adequate vascularisation and oxygen supply in avascular tissues • Loading of physiological forces such as dynamic compression, static loading, shear stress, and fluid flow, in pair with mechanical stability
Ligament	2 weeks to ≥12 months	• Expression of growth factors such as TGF-β, IGF-1, FGF, etc. • Fibroblast activity and collagen type I synthesis • Adequate vascularisation and oxygen supply for angiogenesis • ECM remodelling and collagen deposition • Loading of physiological forces such as tension, compression, and shear, in pair with mechanical stability
Tendon	3 weeks to ≥12 months	• Expression of growth factors such as TGF-β, IGF-1, VEGF, etc. • Inflammatory cytokines and immune responses • Tenocyte activity and collagen production • Adequate vascularisation and oxygen supply for angiogenesis • Loading of physiological forces such as tension, compression, and shear, in pair with mechanical stability to align collagen fibres
Muscle	Days to ≥6 months	• Expression of growth factors such as IGF-1, HGF, FGF, TGF-β, VEGF, etc. • Satellite cell activation and proliferation • Innervation and neuromuscular junction integrity • Adequate vascularisation and oxygen supply for angiogenesis and muscle fibre formation • ECM remodelling and fibrosis control • Loading of physiological forces such as dynamic and static loading, in pair with mechanical stability
Nervous	4 weeks to ≥6 months	• Expression of neurotrophic factors such as NGF, BDNF, GDNF, etc. • Capacity of intrinsic neuron regeneration • Activity of Schwann cells in PNS or glial response in CNS • Axon guidance cues and ECM composition • Inhibition by glial scar in CNS • Adequate vascularisation and oxygen supply for angiogenesis

*BDNF* brain-derived neurotrophic factor, *BMP-2* bone morphogenetic protein-2, *BMP-7* bone morphogenetic protein-7, *CNS* central nervous system, *ECM* extracellular matrix, *EGF* epidermal growth factor, *FGF* fibroblast growth factor, *GDNF* glial cell line-derived neurotrophic factor, *HGF* hepatocyte growth factor, *IGF-1* insulin-like growth factor-1, *MSC* mesenchymal cells, *NGF* nerve growth factor, *PDGF* platelet-derived growth factor, *PNS* peripheral nervous system, *TGF-β* transforming growth factor-beta, *VEGF* vascular growth factor

Therefore, tailoring polymer’s degradation kinetics with tissue regeneration is crucial to allow cell migration and attachment within/on biodegradable scaffolds. The degradation of scaffold materials is necessary to provide a greater area for cell infiltration, attachment, and proliferation to alleviate tissue growth. Too slow degradation will suppress cell proliferation and tissue growth while too fast degradation will remove the platform for cell migration and attachment [30]. An understanding of the remodelling and regeneration of human tissues as well as the knowledge of biodegradable properties of polymeric materials are the key principles in selecting appropriate biodegradable polymers for specific TE applications.

This review article provides a translational view for the selection criteria of synthetic biodegradable polymers in a particular TE field. It covers the overview of biodegradable polymeric scaffolds in TE, followed by the regeneration and remodelling timeline of several types of cells and tissues. The degradation mechanisms employed by different biodegradable polymers were then emphasised. Few common biodegradable synthetic polymers, particularly poly(lactic) acid (PLA), polyvinyl alcohol (PVA), polycaprolactone (PCL), and polyurethane (PU) were elaborated to provide a description of their individual chemical structure, associated degradation mechanism, degradation factors, and timeline. These four types of polymers are among widely used and scientifically established synthetic polymers in biomedical and TE applications, where each of them offers distinct degradation profiles, mechanical properties, and functional versatility [27, 31]. In the later part of the review, an interpretive comprehension of the remodelling and regeneration timeline of the human tissues was aligned with the degradation kinetics of biodegradable polymers to provide an insight for the selection of materials in developing TE scaffolds.

## Degradation mechanisms of biodegradable polymer

The degradation mechanism of biodegradable polymers refers to the fundamental processes of polymer bond dissociation from the initial section into smaller fragments through chemical, physical, and/or biological actions. These mechanisms may act independently or synergistically, depending on the polymer type, chemical structure, and environmental conditions [32]. Biodegradable polymers have received significant attention in recent years, specifically for biomedical and TE applications, due to their degradable capability in acting as temporary tissue replacements [33]. Understanding their degradation mechanisms is crucial for material selection and material design, particularly to optimise functional performances and to predict total tissue replacement in various physiological conditions.

In controlling the biodegradation of synthetic polymers, unstable chemical linkages such as amides, anhydrides, labile esters, orthoesters, etc., can be incorporated in polymer backbones as these bonds are prone to undergo common hydrolytic or enzymatic degradation mechanisms [34]. It generally involves alteration in the physiochemical and mechanical properties of polymers, accompanied by overall mass loss by various mechanisms, and a complete dissociation into degradation by-products. The kinetics of bond cleavage are directly correlated to the type of bonds present in a particular polymeric chain [35, 36]. During active body metabolism, the degraded by-product fragments are transformed into risk-free products and excreted from the body through normal cellular metabolic pathways [37]. Generally, for efficient and tolerable biodegradation, synthetic polymers must have both hydrophobic and hydrophilic moieties, along with amorphous regions in their structure [38]. In addition, surface attributes including wettability, surface charge, polarity, surface roughness, and available chemical moieties on the surface exhibit important roles in aligning degradation targets [22].

**Fig. 1:**
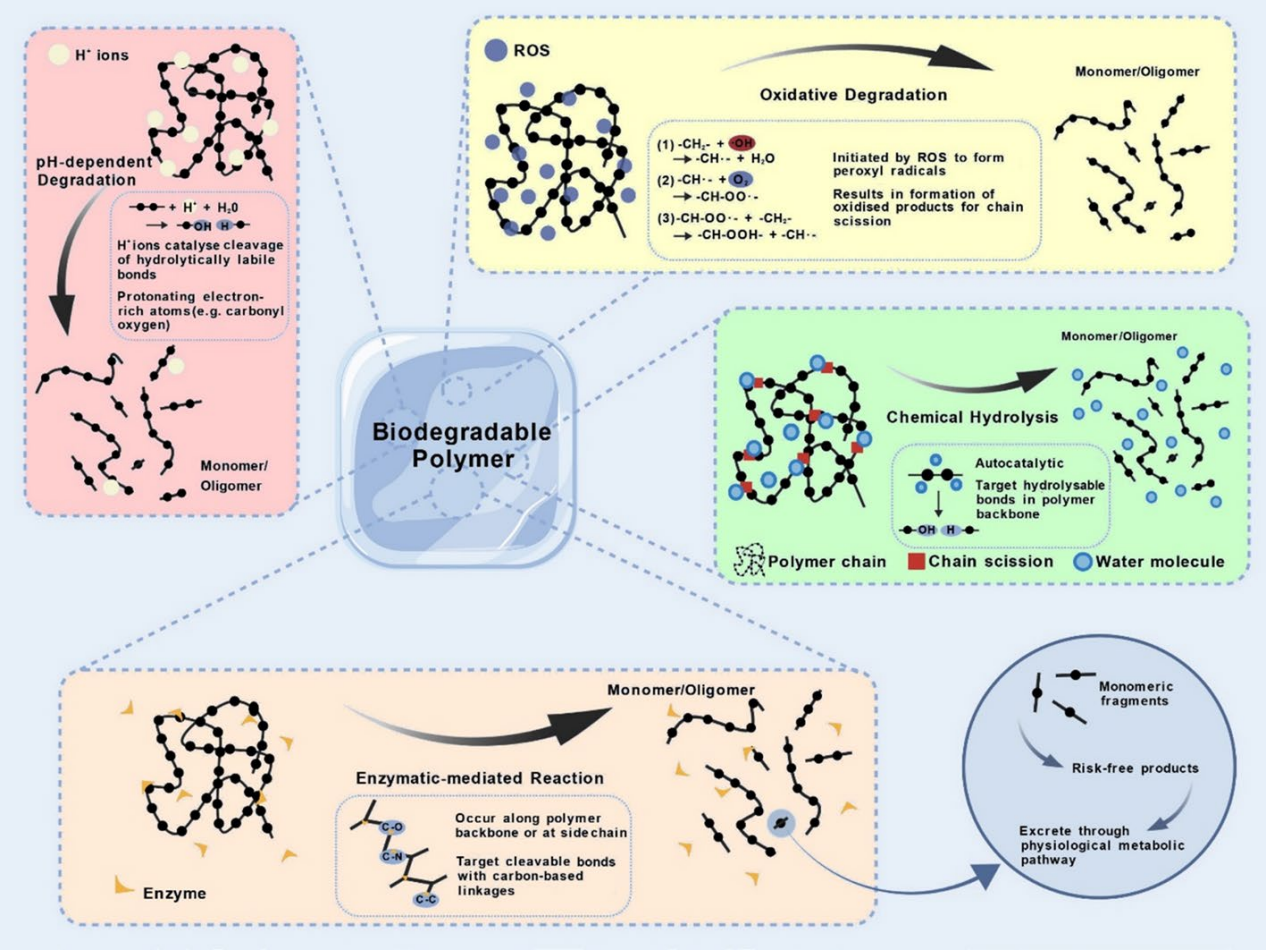
Degradation mechanisms of biodegradable synthetic polymers in physiological environment: chemical hydrolysis, enzymatic-mediated metabolism reaction, oxidative degradation, and pH-dependent degradation (created with BioGPT)

There are various mechanisms in accommodating polymeric degradation. For TE applications, four types of degradation mechanisms are known to be particularly responsible for the degradation of synthetic polymers, including chemical hydrolysis, enzymatic-mediated metabolism reaction, oxidative degradation, and pH-dependent degradation, as shown in Fig. 1. In chemical hydrolysis, susceptible moieties in a polymeric structure undergo autocatalytic scission upon interaction with water molecules and fragment into smaller oligomer and monomeric units without any enzymatic aid [39]. This reaction is susceptible to either bulk polymers through bulk erosion or polymer surfaces through surface erosion that can be identified through mathematical modelling equations [40]. Bulk erosion demonstrates more uniform degradation, where aqueous liquid penetrates faster than the break of chemical bonds [e.g., poly(lactic-*co*-glycolic acid) (PLGA)], while surface erosion is identical to layer-by-layer degradation, where chemical bonds break faster than aqueous liquid penetration (e.g., polyanhydride). Besides, the presence of catalysts or additives may significantly influence the rate of hydrolysis. Few synthetic polymers that are classically associated with the degradation mechanism of chemical hydrolysis include PLA, polyglycolic acid (PGA), PCL, PU, polyanhydrides, and poly(dioxanone).

Enzymatic-mediated metabolism reaction represents another main degradation mechanism of polymeric biodegradable materials in the human physiological environment. Specific enzymes such as esterases, proteases, or lipases are catalysing the cleavage of chemical bonds along the polymer backbone or at the side chain. Their activity is particularly prominent in polymers containing ester, amide, or peptide linkages, where esterases target ester-containing polymers (e.g., polyesters), proteases act on amide or peptide bonds (e.g., polyamides or protein-based materials), and lipases degrade hydrophobic polymers with lipid-like ester groups [41]. The target of cleavable bonds is mainly on carbon-containing linkages. This enzymatic action leads to the gradual breakdown of polymer into smaller fragments that will be naturally metabolised or excreted by the body [42]. The presence and activity of relevant enzymes will further expedite polymer’s degradation rate. To take note, this mechanism is relevant more to natural polymers such as collagen, gelatin, and chitosan. Designing synthetic polymers to degrade in response to specific enzymatic activity is also possible to ensure controlled biodegradation that aligns with tissue regeneration timelines. Among the polymers, PCL can be degraded with the action of lipase and esterase enzymes; PLA and PGA are mainly degraded by chemical hydrolysis but can also be designed towards enzymatic degradation (i.e. PLA degradation through the action of proteinase K and lipase enzymes; PGA degradation through the action of esterase enzyme) while PU is susceptible to urethanase and cholesterol esterase enzymes [40].

Oxidative degradation is another significant mechanism responsible for the breakdown of polymeric materials in the human body. This process involves the interaction of polymers with reactive oxygen species (ROS) such as superoxide anions, hydrogen peroxide, and hydroxyl radicals, which are commonly produced by immune cells such as macrophages and neutrophils during inflammatory responses [43]. Those activated immune cells, particularly macrophages and neutrophils, generate a localised oxidative microenvironment through respiratory burst activity, releasing ROS as part of their innate defence and foreign body response. These species induce oxidative chain scission, backbone oxidation, and surface erosion of polymeric materials [44], thus coupling polymer degradation directly to the intensity and duration of inflammatory responses. As a result, oxidative degradation becomes a biologically regulated process, influenced not only by polymer chemistry but also by immune cell phenotype, activation state, and tissue-specific inflammatory dynamics [43, 44]. Synthetic polymers such as PU and certain PE derivatives are prone to oxidative degradation to support long-term implantations such as vascular grafts and joint implants. In the condition of prolonged inflammatory responses (i.e. infection and chronic inflammation), polymer’s degradation will be accelerated due to greater sources of ROS [45, 46].

In particular, condition that has different cumulation of hydrogen ions (H^+^ ions), pH-dependent degradation is responsible for degrading certain synthetic polymers. Besides, polymeric materials are designed to be dissociated in response to the local acidity or alkalinity of the surrounding physiological environment. In the acidic environments of infected lesions, inflamed tissues, ischaemic or tumour tissues, polymers containing acid- or base-linkages will be easily degraded [47]. Polymers with hydrazone, acetal, ortho ester, and imine bonds are highly responsive towards accelerated hydrolysis when exposed to non-neutral pH levels. For example, poly(ortho esters) and certain polyesters degrade more rapidly under acidic conditions due to proton-catalysed cleavage of their backbone bonds [48]. These smart pH polymers are often explored for the timing of therapeutic release at specific pH-based conditions, valuable in targeting drug delivery. Therefore, understanding the degradation mechanisms of target polymers is essential to navigate the selection and design of polymeric materials that align with the functional requirements of physiological tissues, thus ensuring successful implantation and restoration.

## Synthetic polymers for medical applications

### Polylactic acid (PLA)

Polylactic acid (PLA), also known as polylactide, is a linear aliphatic polyester that has dual advantages of being bio-based and biodegradable [37]. The ester linkages are susceptible to being dissociated into smaller fragments, eventually producing safe degradation products of carbon dioxide, water, and small organic molecules [49]. The competence of low molecular weight PLA to degrade under physiological conditions along with its non-toxicity and biocompatible nature make it a suitable candidate for biomedical applications [50, 51]. The ease of fabrication of PLA allows its synthesisation to be tailored with specific biomedical domains. For instance, most PLA copolymers or blends are used to fabricate devices with desired physicochemical and biological features to achieve controllable biodegradation (i.e. months to years) without obvious provocative reactions [52].

The synthesis of PLA is achieved by polycondensation or ring opening polymerisation (ROP) of lactide, a cyclic compound which is produced due to the condensation or dehydration of two lactic acid molecules. Lactide also can be yielded from bacterial-aided fermentation of lactic acid from plant-based resources such as corn, maize, and sugarcane. The fermentation leads to the production of lactic acids which are converted into lactide monomers and further polymerised into PLA. The monomer, lactic acid (2-hydroxypropionic acid, CH_3_–CHOHCOOH), is a chiral molecule with two optically active enantiomers, L- and D-lactic acid. These enantiomers can form three different types of PLA molecules; L-lactide (PLLA), D-lactide (PDLA), and D, L-lactide (PDLLA); with distinct features through controlled polymerisation processes [50]. Therefore, the overall thermal, physical, mechanical, and crystallinity properties of PLA are dependent upon its molecular weight and stereochemistry. The low molecular weight of PLA contributes to facile biodegradation [53].

#### Degradation mechanisms of PLA

**Fig. 2:**
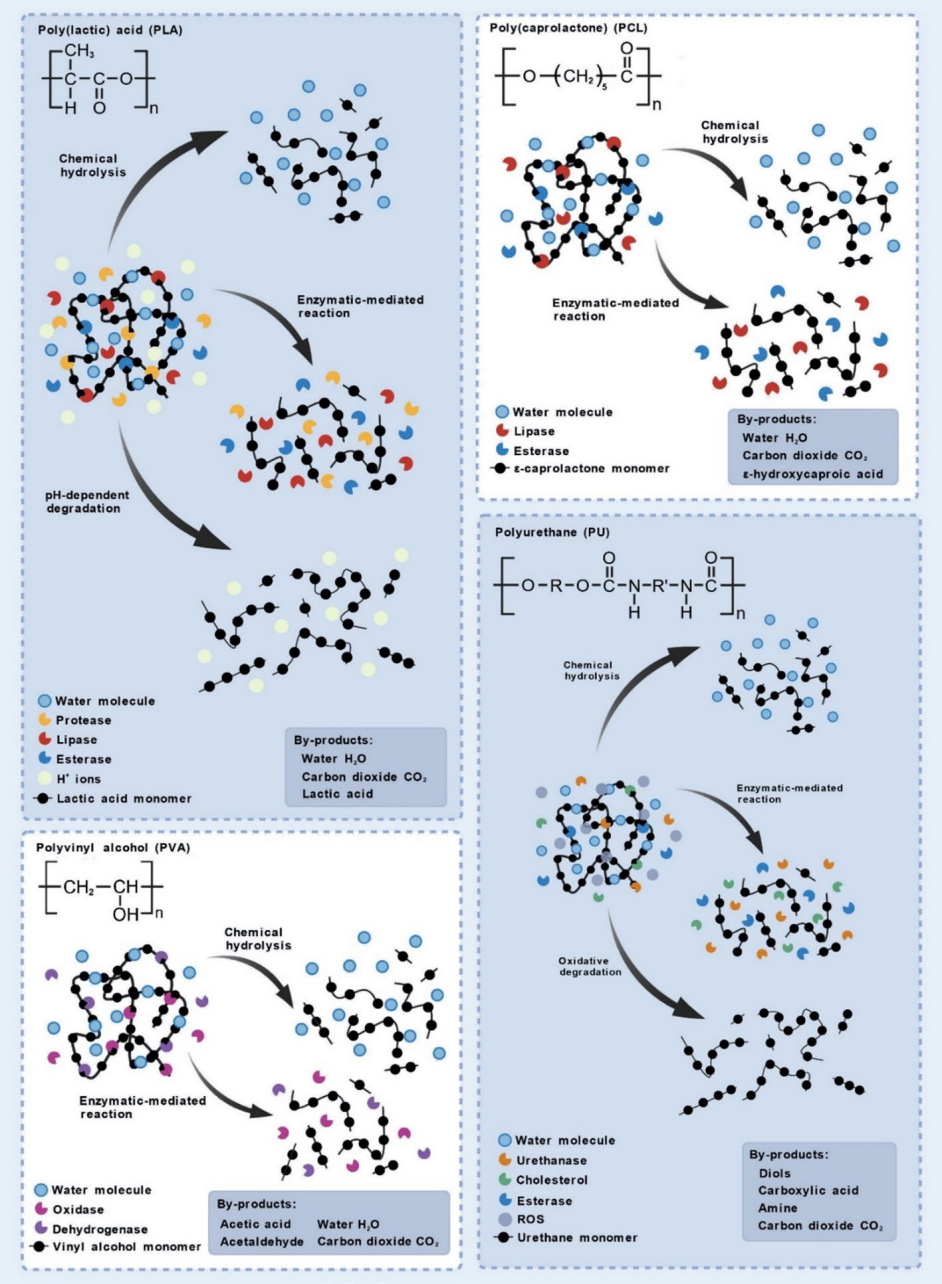
Degradation mechanisms of commonly used synthetic polymers in medical applications including PLA, PVA, PCL, and PU, in relation with dissociation by-products and monomers (created with BioGPT)

Polylactic acid is known to be degraded in a physiological environment through three degradation routes which are hydrolytic chemical hydrolysis, enzymatic-mediation reaction, and pH-dependent degradation mechanisms as illustrated in Fig. 2. As a bulk eroding polymer, PLA is prominently degraded through two phases of non-enzymatic chemical hydrolytic mechanism. In the first phase, during the hydrolytic scission of PLA ester bonds, the long chains will transform into water-soluble low molecular weight oligomers. As hydrolysis proceeds, it will further break the oligomers into monomeric lactic acid units. In the second phase, the degradation products of lactic acid are converted to water (H_2_O) and carbon dioxide (CO_2_) through the Krebs cycle. The products are further metabolised inside the human body to be excreted [54]. The degradation occurs mainly through autocatalysis, end chain, random chain scission, surface erosion, and bulk erosion [55]. However, hydrolysis normally encompasses complex reaction pathways since several different cleavage reactions can happen side by side [52]. Notably, polymer’s degradation involves more chemical events, while erosion is associated with identical physical phenomena such as dissolution and diffusion.

Polylactic acid is also degraded through a pH-dependent degradation mechanism. The accumulation of monomeric lactic acid will create a localised low pH, in both the core of PLA scaffolds and the adjacent environment. The drop in pH gradient will automatically and simultaneously induce more material’s degradation that will be converted into the common PLA by-products of H_2_O and CO_2_ [56]. Despite the hydrolytic chemical hydrolysis and pH-dependent degradation mechanisms, another degradation mechanism of enzymatic-mediation reaction is acting on PLA through commonly three types of enzymes as shown in Fig. 2. Proteases are degrading PLA by hydrolysing its aliphatic domain while lipases and esterases are targeting the ester linkages of PLA. In spite of different routes of degradation, all mechanisms are directing towards the production of monomeric lactic acid, in a subsequent final dissociation into H_2_O and CO_2_, to be metabolised and excreted from the human body.

#### Other factors influencing PLA degradation

In the previous section, degradation mechanisms that are associated with PLA degradation are discussed, which point towards the factors affecting and controlling the degradation. The degradation of PLA proceeds through four distinct phases: (1) the initial loss of mechanical properties governed by the diffusion coefficient of water uptake; (2) the regulation of hydrolysis rate by the amount of water penetrating amorphous regions; (3) the autocatalytic acceleration of hydrolysis caused by carboxylic end-group formation; and (4) the generation of hydrophilic degradation by-products, which ultimately results in substantial weight loss and dissolution of polymer matrix [57]. It can be tuned by altering the stereochemistry, chemical composition, and physical morphology of PLA-based implants, devices, or scaffolds [58, 59]. The hydrolytic cleavage in amorphous PLA regions is significantly facile as compared to the crystalline region where water diffuses linearly into the amorphous regions [60]. Jumat et al. [59] clarified that greater heat is required to mobilise the entangled molecule chains in the higher crystallinity of PLA-based region. The more hydrophobic and crystalline the PLA, the slower the degradation rate. This is due to the less accessibility of water to the hydrolytically unstable ester linkages found in the polymer backbone [61].

For instance, PLA-extruded filaments which possessed 57 ± 2% porosity percentage and 476 ± 52 pore size have degraded 17.6% in Tris–HCl buffer solution containing 0.2 mg/mL of proteinase K and 0.2 mg/mL of sodium azide at 10 days [62]. The blending with calcium carbonate (CaCO_3_) and β-tricalcium phosphate (β-TCP) has produced 68 ± 4% porosity percentage and 518 ± 59 pore size that elevated the filament’s degradation to 22.7%. Similarly, 3D-printed PLA scaffolds degraded in phosphate buffer saline (PBS) over 4 weeks exhibited a baseline degradation of approximately 0–1%, which increased to 2–3% upon the incorporation of 15 wt% magnesium AZ61 alloy [63]. This was accompanied by a shift into a more alkaline degradation environment (pH 8.55), compared to the degradation environment of PLA (pH 7.6). While PLA-casted scaffolds demonstrated much higher degradation in PBS with 20.10% weight loss, which was further increased to 27.4% upon the incorporation of *Pennisetum purpureum* after 40 days of degradation test [64]. The degradation pH remained relatively stable around 7.34–7.43. Another PLA-casted scaffolds immersed in Hank’s balanced salt solution (HBSS) for 7 days showed a weight loss of 1.66% (pH degradation environment of 6.96), which was reduced to 0.93% with the addition of 4% HA and 0.5% graphene oxide (pH degradation environment of 7.78) [65]. The materials showed improved surface wettability from 82.40 ± 4.02° to 73.11 ± 3.66°.

Dehghan-Toranposhti et al. [66] have printed 3D PLA scaffolds with an initial strut-to-strut spacing of 842 µm and surface wettability of 94.1°. They recorded only 0.44% degradation after 10 weeks immersion in PBS which might be due to the supersaturation of degraded materials without environment refreshment. The degradation increased clearly to 3.13% following the addition of 7 wt% manganese (Mn). The PLA/Mn composites with a strut-to-strut spacing of 919 µm have also reduced the surface wettability to 73.4°. Wattanutchariya and Suttiat [67] demonstrated PLA-casted films (wettability of 69.43 ± 0.42°) exhibited a degradation range of ~3–6% after PBS immersion for 90 days. The incorporation of polyethylene glycol (PEG) and 20 wt% bone-derived extracellular matrix (dECM) (wettability of 54.90 ± 0.27°) has enhanced the degradation to ~9–11%. In another study, PLA that has been printed into 3D scaffolds, has degraded ~4–8% in simulated body fluid (SBF) at 12 weeks [68]. The degradation increased significantly to ~17–28% when gelatin/nano-HA was incorporated into the PLA matrix, followed by a coating process with platelet-rich plasma (PRP).

Longer-term degradation study at 20 weeks revealed that PLA scaffolds immersed in PBS exhibited approximately 10% weight loss [69]. Wang et al*.* [70] also showed that 3D-printed PLA scaffolds from their study possessed ~ 8–9% scaffold’s degradation in SBF at 6 weeks, which increased to ~ 12–13% with the incorporation of 35 wt% β-tricalcium phosphate (β-TCP). While an extensive study on nanofibre formation by Bogdanova et al. [71] demonstrated that electrospun PLA nanofibres (diameter of 550 ± 300 nm) have degraded ~ 7–9% over 8 weeks immersion in Fenton’s reagent in vitro. The degradation increased to ~ 11–13% with the inclusion of gelatin (diameter of 380 ± 130 nm). Through in vivo studies, similar authors revealed ~ 10% fibre thinning after one month of implantation while PLA/gelatin nanofibres were thinned up to ~ 30%, indicating greater degradation under physiological conditions. In biomedical applications, PLA are majorly designed towards bone TE that requires a minimum of 6 months of full degradation where the other applications are subjecting general TE area and drug delivery system as summarised in Table 2.

**Table 2:** Previous studies on degradation kinetics of PLA-based materials in various biomedical and TE applications with further estimation on full degradation timeline

Sample type	Experimental procedure	Initial pH condition	Degradation weight loss (%)	Estimated full degradation	Application
PLA-extruded filaments [62]	Immersion in 0.05 M Tris–HCl buffer solution containing 0.2 mg/mL of proteinase K and 0.2 mg/mL of sodium azide at pH 8.6 and temperature of 37 °C. Timeframe: 10 days	Alkaline	17.6	8 weeks	TE
PLA 3D-printed scaffolds [63]	Immersion in PBS at pH 7.6 and temperature of 37 °C. Timeframe: 4 weeks	Neutral	~ 0–1	400 weeks	Bone TE
PLA-casted scaffolds [64]	Immersion in PBS at pH 7.4 and temperature of 37 °C. Timeframe: 40 days		20.10	28 weeks	TE
PLA-casted scaffolds [65]	Immersion in HBSS at pH 7.4 and temperature of 37 ± 0.5 °C. Timeframe: 7 days		1.66	60 weeks	TE, load-bearing implant, clinical devices
PLA 3D-printed scaffolds [66]	Immersion in PBS at pH 7.4 and temperature of 37 ± 0.5 °C. Timeframe: 10 weeks		0.44	43 years	Bone TE
PLA-casted films [67]	Immersion in PBS at pH 7.4 and temperature of 37 °C. Timeframe: 90 days		~ 3–6	214–429 weeks	TE
PLA 3D-printed scaffolds [68]	Immersion in SBF at pH 7.4 and temperature of 37 ± 0.5 °C. Timeframe: 12 weeks		~ 4–8	150–300 weeks	Bone TE
PLA 3D-printed scaffolds [69]	Immersion in PBS at 36.5 °C. Timeframe: 20 weeks		~ 10	200 weeks	Bone TE
PLA 3D- printed scaffolds [70]	Immersion in SBF at 37 °C. Timeframe: 6 weeks		~ 8–9	67–75 weeks	Bone TE
PLA electrospun nanofibres [71]	Immersion in Fenton’s reagent. Timeframe: 8 weeks	Acidic	~ 7–9	89–114 weeks	Suture, orthopaedic device, drug delivery system, and TE

### Polyvinyl alcohol (PVA)

Polyvinyl alcohol is classified as a synthetic polyhydroxy polymer known for its high hydrophilicity, processability, excellent chemical resistance, and biocompatibility [72]. The basic structure of PVA consists of a carbon backbone with hydroxyl groups (-OH) attached to every other carbon atom. Due to the presence of abundant hydroxyl groups, PVA is known to be water-soluble, making this polymer in-match with the hydrophilic moieties of most drugs, bioactive materials, biomolecules, and herbs, thus allowing the formation of a homogenous blending composition [73]. These properties drive its use in biomedical and TE applications, enabling the fabrication of wound dressings, contact lenses, artificial cartilages, and tissue scaffolds [74].

The synthesis of PVA involves the hydrolysis of polyvinyl acetate monomers either through fully or partially hydrolysis procedures [75]. During the hydrolysis process, acetate groups on PVA’s backbone are replaced with hydroxyl groups in the presence of catalysts such as sodium hydroxide (NaOH) or methanol (CH_3_OH), resulting in varying degrees of hydrolysis. The degree of hydrolysis and polymerisation influences the physical properties of PVA such as solubility, crystallinity, and mechanical strength. This feasible alteration allows PVA to be adopted in specific biomedical applications, particularly where properties such as degradation rate, porosity, and elasticity are crucial.

#### Degradation mechanisms of PVA

Polyvinyl alcohol basically degrades through enzymatic-mediation reaction, chemical hydrolysis, and thermal degradation mechanisms. Among these three degradation mechanisms, only the first two routes are associated with PVA degradation in a physiological environment as illustrated in Fig. 2. Certain microorganisms and enzymes such as oxidase and dehydrogenase are actively catalysing PVA through the reaction with hydroxyl groups on PVA chains, leading to chain scission and degradation [76]. The biological degradation of PVA is a random series split of PVA backbone that includes 1,3-glycol structure of two sequential repeating units by a random oxidative dehydrogenation reaction, stimulated by an extracellular secondary alcohol oxidase enzyme [77]. Subsequently, the carbon–carbon bonds are broken, producing smaller alcohol-based fragments (e.g., vinyl alcohol monomers) or oxidised products (e.g., acetaldehyde or acetic acid) [78].

Polyvinyl alcohol is also degraded through a chemical hydrolysis mechanism at a relatively slow rate compared to an enzymatic-mediation reaction due to the stability of its carbon–carbon backbone and the absence of easily hydrolysable linkages such as esters or amides [79]. Although PVA is highly hydrophilic and readily absorbs water, hydrolysis under physiological conditions of neutral pH and a range of body temperature will result in minimal chain scission. This polymer is often chemically modified or blended with more labile and hydrolytically degradable polymers to improve its hydrolytic degradation for TE applications [80]. Among commonly used polymers to be blended with PVA for this purpose are PLA, PGA, PCL, chitosan, gelatin, and starch [81].

#### Other factors influencing PVA degradation

The rate of PVA degradation can be influenced by several factors such as its physical form, crystallinity, porosity, molecular weight, degree of hydrolysis, etc. Partially hydrolysed and less crystalline PVA tend to degrade faster due to increased water permeability and reduced hydrogen bonding [82]. These correlations highlight the importance of balancing polymer concentration and crystalline domains to achieve controlled degradation, suitable for specific tissue engineering and biomedical applications. Alhosseini et al. [83] mentioned that electrospun PVA nanofibres with fibre diameter ranging from 395 to 1105 nm and porosity percentage of 89.3% exhibited rapid degradation, with weight loss reaching approximately 90–100% after 10 days of immersion in PBS. The incorporation of chitosan has reduced the degradation capability, limiting the weight loss to 5–15%. In contrast, freeze-thawed and freeze-dried PVA scaffolds with pore size of 14.1 ± 0.23 μm and porosity percentage of 87.7 ± 0.6% showed slower degradation, with weight loss ranging from ~ 8–15% over 4 weeks in PBS containing 0.4 mg/mL lysozyme [84]. The addition of biphasic calcium phosphate (BCP) reduced the weight loss to ~ 7–12%, and further inclusion of 0.25% carbon nanotubes (CNT) decreased the record to ~ 5–7%. Chen et al*.* [85] stated that porous freeze-dried PVA scaffolds immersed in PBS for 7 days experienced 11.15% weight loss, which increased slightly to 11.59% (low molecular weight) and 13.28% (medium molecular weight) upon the incorporation of chitosan.

In another study by Fathi et al*.* [86], electrospun PVA fibres with diameter of 842 ± 205 nm, pore size of 1.33 ± 0.49 μm, and porosity percentage of 74 ± 3.9% showed moderate degradation (~ 20–30%) over 14 days. The blending with chitosan produced the fibres with 1070 ± 340 nm diameter, 0.9 ± 0.31 μm pore size, and 52 ± 4.7% porosity percentage that caused the degradation to sharply increase to ~ 70%. Similarly, the incorporation of both chitosan and silk raised the weight loss to ~ 50–60%, with structural changes of 1200 ± 321 nm fibre diameter, 2.2 ± 0.9 μm pore size, and 68 ± 4.4% porosity percentage. Lan et al*.* [87] measured that freeze-thawed PVA hydrogels with pore size of 34.95 ± 15.6 μm showed weight loss of ~ 25–30% over 4 weeks in lysozyme-containing PBS. The degradation increased significantly to 60–70% with the addition of collagen at 1:2 PVA:Col-II ratio (initial pore sizes of 42.77 ± 15.1 μm). While PVA scaffolds cross-linked with 1-ethyl-3-(3-dimethylaminopropyl)-carbodiimide (EDC) and N-hydroxysuccinimide (NHS) lost ~ 10% of their weight over 16 days in PBS, the degradation increased dramatically to ~ 100% with the incorporation of chitosan [88].

In another study by Dorkhani et al*.* [89], non-crosslinked freeze-thawed PVA hydrogels showed fast degradation, with a weight loss of ~ 90–100% after only 4 days in PBS. To improve the stability of PVA hydrogel, Mawad et al*.* [90] have cured 83% and 98% hydrolysed PVA hydrogels with UV and both underwent 100% degradation in PBS at 70 days and 130 days, respectively. Three-dimensional printed bioink scaffolds made of 15% w/v PVA were found degraded by ~ 10% over 28 days in a high glucose cell culture medium [91]. The incorporation of 10% alginate has increased the degradation to ~18–20%. While PVA freeze-thawed hydrogels immersed in SBF for 5 weeks showed a decline in degradation with the increasing of PVA concentrations; 2% PVA (45% weight loss), 5% PVA (38% weight loss), and 10% PVA (34% weight loss) [92]. The addition of 2% bioglass to the 10% PVA has reduced the weight loss to 27%. The utilisation of PVA in fabricating scaffolds and hydrogels is mostly concentrated on skin TE that requires days to weeks of degradation. Minorly, it is dedicated to bone, cartilage, neural, and cornea applications as summarised in Table 3.

**Table 3:** Previous studies on degradation kinetics of PVA-based materials in various biomedical and TE applications with further estimation on full degradation timeline

Sample type	Experimental procedure	Initial pH condition	Degradation weight loss	Estimated full degradation	Application
PVA electrospun nanofibres [83]	Immersion in PBS at pH 7.4 and temperature of 37 °C. Timeframe: 10 days	Neutral	~ 90–100%	10–11 days	Neural TE
PVA freeze-thawed and freeze-dried scaffolds [84]	Immersion in PBS that contained 0.4 mg/mL lysozyme at 37 °C. Timeframe: 4 weeks		~ 8–15%	27–50 weeks	Bone TE
Porous PVA freeze-dried scaffolds [85]	Immersion in PBS at pH 7.0 and temperature of 37 °C. Timeframe: 7 days		11.15%	63 days	TE
PVA electrospun fibres [86]	Immersion in PBS at 37 °C. Timeframe: 14 days		~ 20–30%	47–70 days	Skin TE, skin substitute
PVA freeze-thawed hydrogels [87]	Immersion in PBS that contained 0.4 mg/mL lysozyme at 37 °C. Timeframe: 4 weeks		~ 25–30%	13–16 weeks	Cartilage TE
PVA-casted scaffolds with further cross-linking with EDC-NHS [88]	Immersion in PBS at pH 7.4 and temperature of 37 °C. Timeframe: 16 days		~ 10%	160 days	Corneal TE
PVA freeze-thawed hydrogels [89]	Immersion in PBS at 37 °C. Timeframe: 4 days		~ 90–100%	4–5 days	Parenchymal haemorrhage
PVA UV-cured hydrogels; 83% hydrolysed, high functional group density [90]	Immersion in PBS at pH 7.4 and temperature of 37 °C		100% weight loss; timeframe: 70 days	70 days	Drug delivery, liquid injection
PVA 3D-printed bioink scaffolds [91]	Immersion in high glucose Dulbecco’s modified eagle medium (DMEM) containing foetal bovine serum, penicillin, streptomycin, and Fungizone, cultured in 5% CO_2_ and temperature of 37 °C. Timeframe: 28 days		~ 10%	40 weeks	Cartilage TE
PVA freeze-thawed hydrogels [92]	Immersion in SBF at pH 7.4 and temperature of 37 °C. Timeframe: 5 weeks		2% PVA: 45% 5% PVA: 38% 10% PVA: 34%	2% PVA: 11 weeks 5% PVA: 13 weeks 10% PVA: 15 weeks	Skin TE

### Polycaprolactone (PCL)

Polycaprolactone (PCL) is one of the widely explored synthetic linear polyesters, generally prepared by a ring opening polymerisation of ε-caprolactone [93]. The existence of ester linkages between carboxyl and hydroxyl groups in the PCL polymeric chain renders its semicrystalline and hydrophobic nature, further tailoring its biodegradability [94]. Polycaprolactone is often adopted as a homopolymer or as a copolymer with other biodegradable monomers (e.g., lactide, glycolide, trimethylene carbonate, or polydioxanone) to tailor its resorption time. Its promising attributes such as availability, stable structure, easy processing, superior mechanical properties, long-term degradation profile, and biocompatibility cause this polymer to be demanded for TE and drug delivery applications [95, 96]. Polycaprolactone can be processed through various fabrication techniques, such as electrospinning, 3D printing, and solvent casting, that is often combined with other polymers or compounds to enhance its biological performances [97, 98].

The polymerisation of ε-caprolactone monomers is typically initiated by metal catalysts such as stannous octoate (Sn(Oct)_2_) in the presence of an alcohol initiator [99]. Alcoholic initiators such as methanol, ethanol, or diols determine the chain length and end-group functionality of the resulting polymer. This polymerisation technique allows for precise tuning of molecular weight and polymer architecture by controlling the ratio of monomer to initiator and catalyst. The mild reaction conditions and scalability of this synthesis process make PCL an attractive option for industrial-scale production and biomedical applications that require high-purity and customisable polymeric materials [100].

#### Degradation mechanisms of PCL

Polycaprolactone can degrade over a time span of a few months to years depending upon the structural composition, mechanical properties, and environmental conditions. Generally, in the human body, the degradation of PCL follows two consecutive degradation mechanisms of chemical hydrolysis and enzymatic-mediated reaction as shown in Fig. 2. At first, ester bonds in the PCL composition are hydrolysed, producing ε-hydroxycaproic acid oligomers and monomers. This process is slow as PCL is hydrophobic and possesses high crystallinity which limits the ability of water to penetrate [93]. In the human body, macrophages (i.e. a type of white blood cell) will subsequently initiate intracellular degradation through the process of phagocytosis where polymer fragments or residues known as foreign subjects are engulfed and internalised into phagosomes [101]. The fusion of phagosomes with lysosomes that contain acidic digestive enzymes will form a biological structure called phagolysosomes that accelerate the cleavage of polymer chains. This intracellular degradation markedly reduces the polymer’s molecular weight and compromises its structural cohesion, leading to a progressive loss of mechanical strength [101]. Over time, the breakdown products are further metabolised or expelled from the cells, thus completing the resorption process. However, the rate of hydrolytic degradation is influenced by multiple factors including its chemical structure, molecular weight, geometry, wettability, temperature, pH, and enzymatic activity. Based on its chemical structure, PCL degrades inside the human body over a span of two to four years [100].

The enzymatic-mediated degradation of PCL occurs through the cleavage of ester bonds in the polymer backbone by specific enzymes, particularly lipases and esterases [93, 102]. These enzymes catalyse the hydrolysis of ester linkages, leading to the formation of lower molecular weight oligomers and eventually ε-hydroxycaproic acid. Unlike slow hydrolytic degradation, enzymatic-mediated degradation may act robustly, especially in a biological environment where enzymes are present [103]. This degradation mechanism is typically more effective in the amorphous regions of PCL, where the polymer chains are less densely packed and more accessible. The surface erosion is common in enzymatic-mediated degradation, as enzymes act primarily at polymer surfaces [104, 105]. In TE applications, enzymatic degradation plays a more pronounced role, where enzymes from bodily fluids or surrounding tissues can facilitate the breakdown of PCL. This mechanism is particularly valuable in designing biodegradable scaffolds, sutures, or drug delivery systems that require a controlled and biologically responsive degradation profile.

#### Other factors influencing PCL degradation

The degradation rate of PCL is influenced by several key factors that affect both hydrolytic and enzymatic degradation mechanisms. Molecular weight plays a significant role, where lower molecular weight PCL degrades faster due to shorter polymer chains and greater end-group accessibility [106]. Crystallinity is another critical factor where dense crystalline lamellae have low free volume that reduce the polymer’s chain mobility, thus limiting water absorption and enzyme access [106, 107], while amorphous regions are more readily available for hydrolysation and enzyme penetration. As a result, degradation typically proceeds by preferential cleavage of amorphous chains. Surface area and porosity also impact the degradation rate of PCL, where greater porosity will contribute to higher surface area for the action of surface erosion degradation [108].

In the previous study, cast PCL films exhibited significantly higher degradation of 20–30% within 23 days [109]. Whereas the combination with PEG to form PCL–PEG–PCL triblock copolymers reduced the weight loss to 0–20%, due to altered crystallinity and water interaction. For bulkier scaffolds such as PCL bone bricks, the initial pore size of 333–389 μm and porosity above 50% enabled a moderate 5.07% degradation within 5 days [110]. However, by incorporating 20 wt% bioglass, the degradation dramatically increased to approximately 90%, suggesting the critical role of bioglass in re-arranging the molecular structure of PCL, thus accelerating hydrolysis and dissolution processes. In another study by Åkerlund et al*.* [111], cylindrical PCL filaments showed negligible degradation, 0.6% in 28 days. The blending of 59.5 wt% PLA and 15 wt% HA boosted the weight loss to 60–65%, demonstrating the synergistic effect of blending and composite formulation in tailoring degradation behaviour. Electrospun PCL nanofibres with an average 1.76 ± 0.44 μm fibre diameter and 8.73 ± 2.28 μm pore size demonstrated a relatively slow degradation rate of approximately 4–5% over a prolonged period of 28 weeks [112]. However, when fabricated into a trilayered structure incorporating PVA–PCL–PVA, the weight loss has enhanced to 12–14%, likely due to increased hydrophilicity and water uptake. A study by Khrisna et al*.* [108] showed that electrospun PCL nanofibres with fibre diameter of 1.73 ± 0.35 μm and porosity of 27.09 ± 4.07% have produced 2.5–3.0% degradation at 60 days of analysis due to their inherent hydrophobicity (115.03 ± 3.69°). Yet, the blending with oestrogen, followed by surface functionalisation with polydopamine (PDA), has increased the porosity to 47.21 ± 5.78% and lower the hydrophobicity to 32.25 ± 2.15°, which led to an increment in degradation (11.29 ± 2.78%).

Khoshnood et al. [113] found that 3D-printed PCL scaffolds with surface wettability of 72.4° showed only 1.51% weight loss in 4 days, whereas polyethyleneimine (PEI) coating has enhanced the hydrophilicity to 43.4° and increased the degradation to 5.77%. Magnetic PCL scaffolds fabricated through freeze-drying demonstrated a baseline degradation of 1.8–2.0% at 8 weeks, which raised to 9.5–10% upon the addition of 10 wt% nHA [114]. While highly hydrophobic electrospun nanofibres with contact angle of 133 ± 1.3°, the degradation remained minimal (0–3% over 12 weeks) [115]. Following an acid treatment with potassium permanganate for 48 h, the weight loss exceeded 25%, indicating the effect of chemical oxidation to increase surface reactivity and degradation kinetics. Moreover, electrospun nanofibres containing 60 wt% polyethylene ether (PEE) exhibited a notable increased degradation from 4.97 to 21.04% within 28 days due to improved hydrophilicity [116].

Bead-free electrospun nanofibres, characterised by very fine diameters (175 ± 0.95 nm) and high porosity (87.33 ± 3.56%), have degraded 1.35% in 2 weeks [117]. The introduction of chitosan and 25 μM curcumin enhanced the degradation to 25.76 ± 4.90%. These modifications also significantly increased the fibre diameter and porosity to 526 ± 0.95 nm and 84.27 ± 5.32%, respectively. Polycaprolactone has been widely adopted in various TE applications, with particular emphasis on bone TE as presented in Table 4. Minor studies are utilising PCL for skin TE.

**Table 4:** Previous studies on degradation kinetics of PCL-based materials in various biomedical and TE applications with further estimation on full degradation timeline

Sample type	Experimental procedure	Initial pH condition	Degradation weight loss (%)	Estimated full degradation	Application
PCL-casted film [109]	Immersion in 5 M NaOH at temperature of 37 °C. Timeframe: 23 days	Alkaline	~ 20–30	11–16 weeks	TE
PCL bone bricks [110]	Immersion in 5 M NaOH at pH 14 and temperature of 20 °C. Timeframe: 5 days		5.7	13 weeks	Bone TE
PCL cylindrical filaments [111]	Immersion in 0.1 M NaOH at temperature of 37 °C. Timeframe: 28 days		0.6	12.8 years	Bone TE
PCL electrospun nanofibres [112]	Immersion in PBS at pH 7.4 and temperature of 37 °C, shaken at 100 rpm. Timeframe: 28 weeks	Neutral	~ 4–5	10.7–13.4 years	TE
PCL electrospun nanofibres [108]	Immersion in deionised water at temperature of 37 °C. Timeframe: 60 days		~ 2.5–3.0	5.5–6.6 years	Bone TE
PCL 3D-printed scaffolds [113]	Immersion in PBS containing 0.5 mg/mL proteinase K at temperature of 37 °C. Timeframe: 5 days		1.51	47 weeks	Bone TE
Magnetic PCL freeze-dried scaffolds [114]	Immersion in PBS at pH 7.2 and temperature of 37 °C. Timeframe: 8 weeks		~ 1.8–2.0	7.7–8.5 years	Biomedical
PCL electrospun nanofibrous [115]	Immersion in SBF containing 1% H_2_O_2_ at temperature of 37 °C. Timeframe: 12 weeks		~ 0–3.0	> 7.7 years	TE
PCL electrospun nanofibres [116]	Immersion in PBS at 37 °C. Timeframe: 28 days		4.97	1.5 years	TE
PCL electrospun nanofibres [117]	Immersion in SBF at pH 7.3 and temperature of 37 °C. Timeframe: 2 weeks		1.35	3 years	Skin TE

### Polyurethane (PU)

Medical grade PU is one of the synthetic polymers, commonly used in TE field in the form of foams, membranes, and films. It consists of organic units which are joined by carbamate compounds. Most PUs are classified as thermosetting polymers that do not melt when heated while thermoplastic PUs are also available in the market [118, 119]. This polymer has superior mechanical properties compared to other synthetic polymers and possesses long-term degradation stability [120]. It is composed of soft and hard segments where its structure can be chemically synthesised to meet the specific requirements of various tissues, ranging from soft (e.g., skin, cardiac) to load-bearing (e.g., bone) applications [119, 121].

Medical grade PUs are typically synthesised using biocompatible aliphatic diisocyanates or polyisocyanate, polyester or polyether polyols, and chain extenders that contain hydrolysable or enzymatically cleavable linkages [119]. These chain extenders allow for controlled degradation under physiological conditions. The properties of PU are primarily determined by the interaction between its isocyanate and polyol components. When PCL-based polyols are used, the PU composite is tuned into soft and elastic structures due to the flexible nature of PCL segments. Its elasticity and structural integrity make it dominant to be used as scaffolds, vascular grafts, and wound healing materials, where mechanical resilience and gradual resorption are essential [122, 123].

#### Degradation mechanisms of PU

Medical grade PU is degraded through mainly chemical hydrolysis, enzyme-mediated reactions, and oxidative degradation mechanisms, producing non-toxic degradation products (Fig. 2). In primary hydrolytic degradation, ester bonds are hydrolysed into subsequence degradation products of α-hydroxy, urethane, and urea units with terminal acid groups [124]. Water molecules dissociate these hydrolysable linkages, leading to chain scission, molecular weight reduction, and eventual disintegration of PU. Further degradation of urethane and urea units will direct into the release of free polyamines that depends on the initial composition of monomers [41].

The enzymatic degradation of PU occurs through the action of biological enzymes that cleave specific bonds within the polymer structure, particularly ester, urethane (carbamate), and sometimes urea linkages [125]. This mechanism is especially relevant in physiological environments, where enzymes such as esterases, lipases, proteases, and cholesterol oxidases are naturally present and can interact with PU-based biomaterials [124]. The soft segments of PU, especially those derived from aliphatic polyesters, are prone to enzymatic attack due to their flexible, accessible structure, and the presence of ester groups [126]. Enzymes catalyse the hydrolysis of ester bonds, leading to the formation of oligomers and small molecules such as diols and carboxylic acids. The hard segments, typically composed of urethane or urea groups formed from biocompatible diisocyanates, are more resistant but can also be degraded slowly under prolonged enzymatic exposure [127, 128].

The oxidative degradation of PU involves the breakdown of polymer backbone through reactions with ROS, such as superoxide anions (O₂⁻), hydrogen peroxide (H₂O₂), and hydroxyl radicals (•OH) [46]. These ROS are commonly generated in the physiological environment as part of the body’s immune response, particularly by activated macrophages and neutrophils at the site of implantation. The functional groups in PU that are most susceptible to oxidative attack include ether, urethane, and aromatic segments [129, 130]. The ROS induce chain scission, crosslinking, or backbone oxidation, which result in the formation of carbonyl-containing degradation products such as aldehydes, ketones, and carboxylic acids [131, 132]. These reactions lead to a gradual loss of mechanical strength, increased brittleness, and eventual fragmentation of PU material. The rate and extent of oxidative degradation depend on the chemical composition of PU, whether it contains aliphatic and/or aromatic biocompatible diisocyanates [133]. Aliphatic PUs have a tendency to slow degrade compared to aromatic PUs that are more susceptible to oxidative cleavage.

#### Other factors influencing PU degradation

The degradation rate of PU is influenced by several structural factors including the polymer’s hydrophilicity, crystallinity, crosslinking density, and surface area. Additionally, pH may accelerate the hydrolytic process to catalyse ester bond cleavage more rapidly. However, the pH-dependent degradation mechanism is not effectively a role in degrading PU as much as PLA since the degradation products of PU are weak carboxylic acids that can be buffered by physiological fluids [134]. Interestingly, highly porous or nanostructured PU materials with large surface areas are more susceptible to all hydrolytic, enzymatic, and oxidation attacks.

Polyurethane electrospun nanofibres exhibited varying degradation rates depending on their structural and compositional modifications. Polyurethane cast films exhibited slower degradation, with a range of 2.7–5.0% at 12 months, although the addition of macrodiols could increase the degradation to approximately 4.64% [135]. One formulation with a pore size of 1.9 ± 0.4 µm, porosity of 55.7 ± 4.0%, and hydrophobicity of 111.1 ± 0.7° showed a baseline degradation of approximately 4–5% over 60 days [136]. However, when chitosan and 1.0% elastin were incorporated, the weight loss increased substantially to 14–28%. A related formulation with improved porosity (63.3 ± 6.4%) and lower hydrophobicity (67.1 ± 8.8°) further demonstrated the influence of these physicochemical parameters on degradation kinetics. Another PU electrospun membrane, with nanofibre diameter of 0.87 ± 0.06 µm, showed a higher degradation range of 15–18% after 60 days, which increased to 18–30% when chitosan and elastin were added [20]. Polyurethane foams degraded much more slowly, with a reported degradation of 80% over a span of three years [137]. However, this rate significantly increased to 92% upon the incorporation of poly(DL-lactide-co-ε-caprolactone). Ahmad Tarmizi et al*.* [138] described electrospun PU-grafted with 2-hydroxyethyl methacrylate (HEMA) showed a much slower degradation, with weight loss reduced to 0.5–1.5% compared to the initial PU degradation of 13–15% at 84 days.

For PU-injected moulding films, a moderate degradation of 10–13% was observed at 10 weeks [139]. The inclusion of 40% PLGA not only increased the pore diameter but also raised the weight loss to 22–25%. Selvaras et al*.* [140] showed that electrospun PU fibres loaded with chitosan nanoparticles showed a mild increase in degradation from 3–4% to 4–5% over 60 days. While Tsai et al. [141] elaborated PU 3D freeze-dried scaffolds with extremely high porosity of 97.2 ± 0.1% and pore diameter of 154.4 ± 28 µm have degraded approximately 5% after 28 days. In another study by Nabipour et al*.* [142], electrospun PU with hydrophobicity of 107.6 ± 0.6° had a weight loss of ~ 3% after 8 weeks. The incorporation of multi-walled carbon nanotubes (MWCNTs) reduced the hydrophobicity to 99.8 ± 1.4° and raised the degradation to approximately 9%, indicating the catalytic role of nanomaterials in enhancing PU degradation. Vakil et al*.* [143] produced PU-moulded foams with a large pore size of 1100 ± 300 µm that exhibited different degradation behaviours in vitro and in vivo. Through the in vitro analysis, PU’s degradation ranged from 0 to 20% over 12 weeks, while through the in vivo analysis, PU’s degradation was significantly higher at 30–40% within the same duration. Notably, the inclusion of 30% diethylene glycol (DEG) and nitrilotriacetic acid-modified DEG (NTA-DEG) drastically reduced PU’s degradation to approximately 0% for the in vitro, but increased the degradation to 60–70% for the in vivo. Most of this research are addressing vascular and neural TE applications which attempted for blood contacting device as shown in Table 5.

**Table 5:** Previous studies on degradation kinetics of PU-based materials in various biomedical and TE applications with further estimation on full degradation timeline

Sample type	Experimental procedure	Initial pH condition	Degradation weight loss	Estimated full degradation	Application
PU-casted films [135]	Immersion in PBS and additional 0.02 wt% of sodium azide (NaN_3_) at temperature of 37 °C. Timeframe: 12 months	Alkaline	~ 2.7–5.0%	~ 19–37 years	Biomedical engineering
PU electrospun nanofibres [136]	Immersion in PBS at pH 7.4 and temperature of 37 °C, shaken at 100 rpm Timeframe: 60 days	Neutral	~ 4–5%	~ 3.3–4.1 years	Vascular TE
PU electrospun nanofibres [20]	Immersion in PBS at pH 7.4 and temperature of 37 °C, shaken at 100 rpm Timeframe: 60 days		~ 15–18%	~ 1–1.1 years	Vascular TE
PU foams [137]	Immersion in Sorensen buffer at pH 7.4 and temperature of 37 °C, shaken at 100 rpm Timeframe: 3 years		80%	3.75 years	TE
PU electrospun nanofibres [138]	Immersion in PBS with 1% of penicillin and streptomycin at temperature of 37 °C Timeframe: 84 days		~ 13–15%	~ 1.5–1.8 years	Artificial blood contracting medical devices
PU-injected moulding film [139]	Immersion in PBS at temperature of 37 °C, shaken at 90 rpm. Timeframe: 10 weeks		~ 10–13%	~ 1.5–1.9 years	Neural TE
PU electrospun nanofibres [140]	Immersion in PBS at temperature of 37 °C, shaken at 100 rpm. Timeframe: 60 days		~ 3–4%	~ 4.1–5.5 years	Vascular TE
PU 3D freeze-dried scaffolds [141]	Immersion in enzymatic papain-digested buffer at pH of 7.4 and temperature of 37 °C. Timeframe: 28 days		~ 5%	~ 1.5 years	Cartilage TE
PU electrospun nanofibres [142]	Immersion in PBS at temperature of 37 °C, shaken at 100 rpm. Timeframe: 8 weeks		~ 3%	~ 5.1 years	Neural TE
PU-moulded foams [143]	Immersion in 3% H_2_O_2_ at temperature of 37 °C Timeframe: 12 weeks	Acidic	In-vitro: ~ 0–20% In-vivo: ~ 30–40%	In-vitro: ≥ 59.9 weeks In-vivo: ~ 30–40 weeks	Biomedical and TE

### Interpretive comprehension on tailoring degradation kinetics of synthetic polymers with tissue regeneration timeline

Designing biodegradable synthetic polymer-based scaffolds for biomedical and TE applications requires precise synchronisation between polymer’s degradation kinetics and the intrinsic regeneration of physiological tissues. Different tissues have vastly different healing and regeneration duration—the cornea may remodel from days to months while the skin may remodel in as minimum as 2 weeks, the bone often requires 6–12 weeks, while the cartilage, ligament, vascular, and neural regenerations can extend from several months to over a year. An optimal scaffold should maintain its mechanical integrity and act as a temporary ECM until the regenerating tissues can independently bear functional loads. A mismatch between degradation and regeneration can be detrimental where premature degradation will remove the physical framework needed for cell migration, adhesion, and ECM deposition; whereas, overly slow degradation will restrict spaces for new tissue growth, further triggering chronic inflammation.

**Fig. 3:**
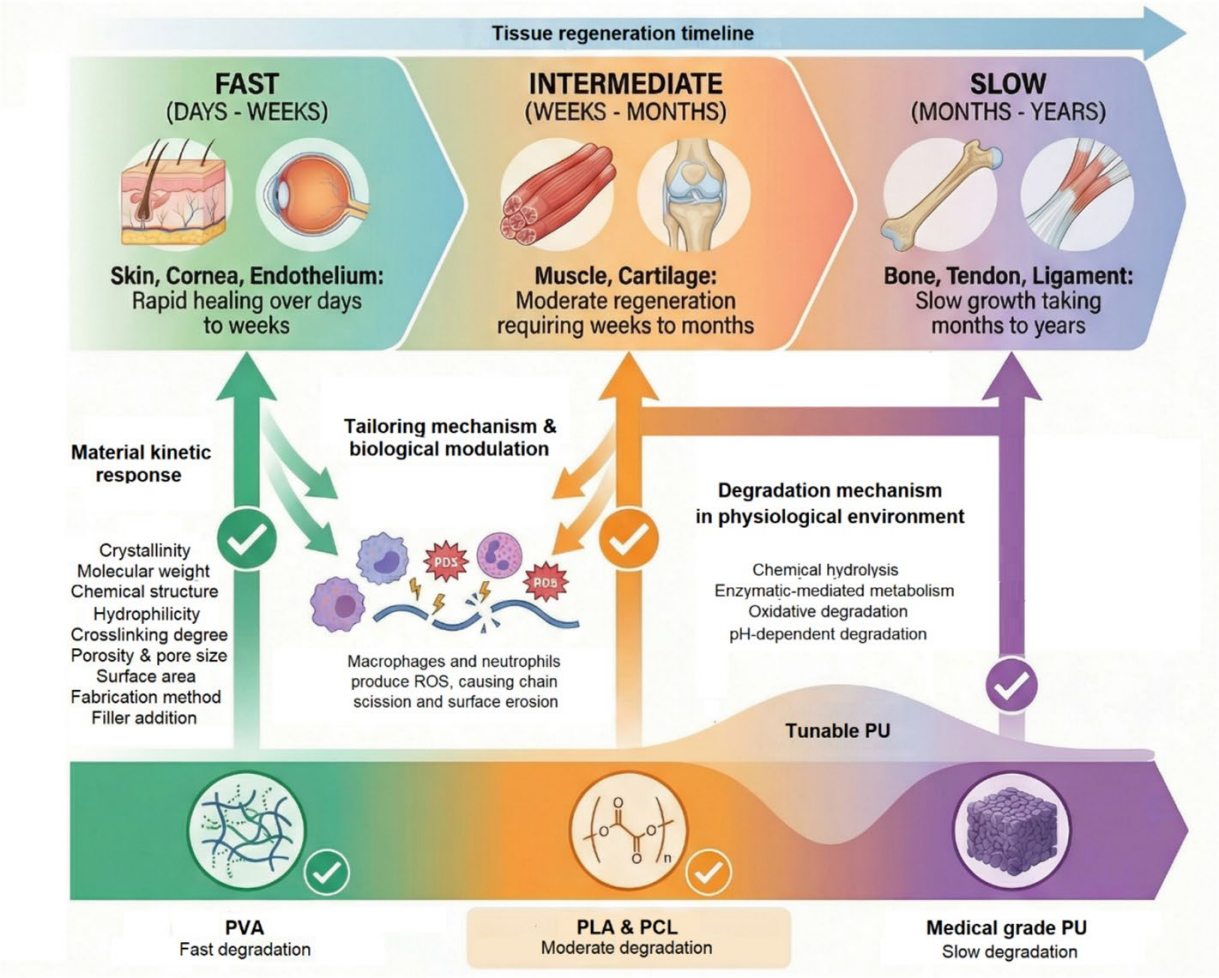
Tailoring of degradation kinetics of synthetic polymers with tissue regeneration timeline (Created with NotebookLM)

Synthetic biodegradable polymers such as PLA, PVA, PCL, and PU offer versatility in degradation control through the adjustment of chemical structure, stereochemistry, crystallinity, molecular weight, surface chemistry, porosity, and blending with other degradable components. For example, PLA degrades primarily through a hydrolytic scission, with the timeline ranging from weeks to years. Thus, it is suitable for biomedical and TE applications, spanning from fast-healing soft tissues to slow-remodelling bone. While PVA is known to be highly hydrophilic but hydrolytically stable, it degrades more rapidly when blended with labile polymers or enzymatically active components. This polymer aligns well with short-to-intermediate healing tissues such as the skin and the cornea. The hydrophobicity and high crystallinity of PCL and PU yield degradation profiles of several years unless modified, making them favourable for long-term implantations such as load-bearing bone, cartilage, ligament, neural, and vascular implantations. In addition, the tunable properties of PU allow for gradual degradation through hydrolytic, enzymatic, and oxidative degradation mechanisms, supporting applications that require mechanical resilience over extended healing periods, including vascular grafts and cartilage regeneration. Figure 3 shows the tailoring of degradation kinetics of synthetic polymers with tissue regeneration timeline.

In association with the physiological environment, there are four types of degradation mechanisms commonly involved, including chemical hydrolysis, enzymatic-mediated reaction, oxidative degradation, and pH-dependent degradation. These degradation mechanisms can be activated through singular or multiple actions to achieve the desired degradation kinetics. Chemical hydrolysis offers predictable bulk erosion while the enzymatic-mediated reaction enables site-specific or biologically triggered breakdown. Oxidative degradation becomes relevant in inflammatory or vascular environments, and the pH-dependent mechanism allows for localised, stimulus-responsive resorption. Ultimately, a scaffold design must integrate the knowledge of both material science and tissue physiology. This involves predicting polymer degradation behaviour in physiological conditions, understanding biochemical and mechanical requirements of the target tissues during healing, and applying fabrication strategies such as the design of scaffold architecture that maintains an optimal balance between structural support and the material’s degradation. Each factor requires extensive and deep comprehension for further review. Such tailoring ensures that scaffold disappearance coincides with functional tissue restoration, translating biomaterial design into effective clinical outcomes.

**Fig. 4:**
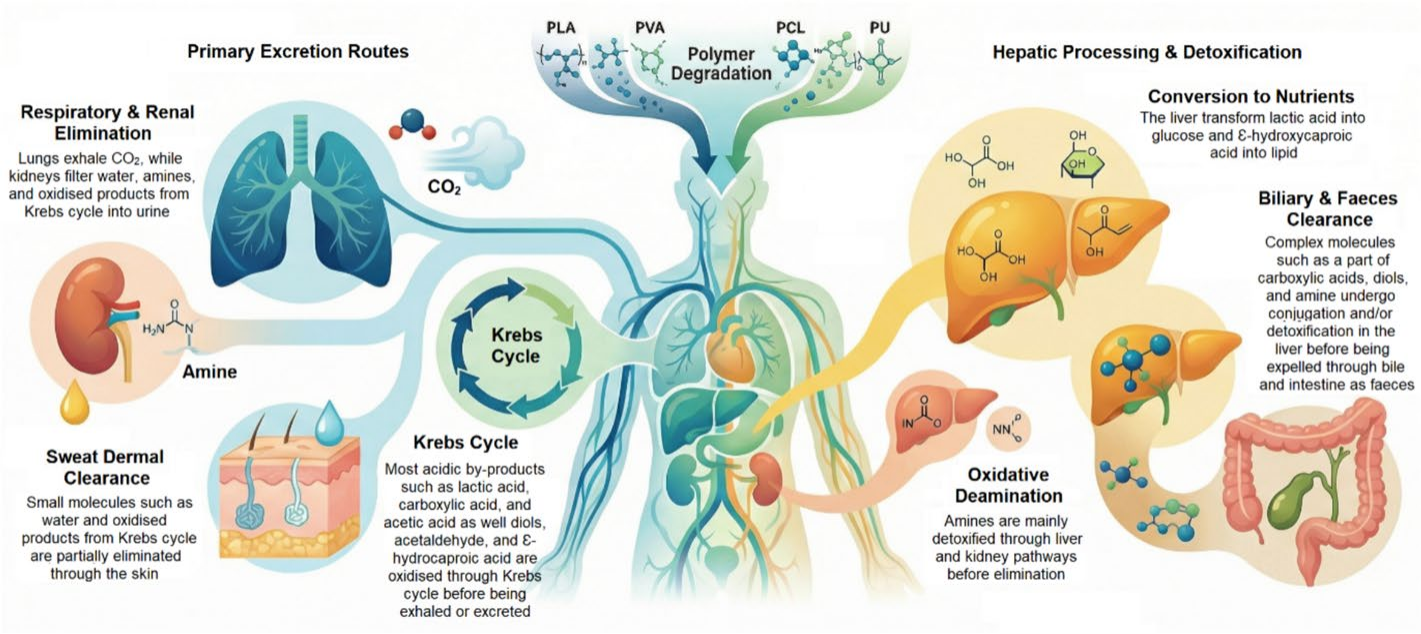
Clearance routes for the degradation by-products of PLA, PVA, PCL, and PU that involve various functional organs such as kidney, lung, liver, and skin (created with NotebookLM) [54–56, 78, 79, 93, 103, 143, 144]

Apart from degradation mechanisms, degradation by-products, as illustrated in Fig. 2, should be cleared efficiently from the human body to avoid local accumulation, inflammation, and toxicity. While the by-products are being eliminated, the polymers should support tissue regeneration within the tissue regeneration timeline. Figure 4 presents the frequent clearance routes for the degradation by-products of PLA, PVA, PCL, and PU. Water and carbon dioxide are two dominant by-products from the degradation of synthetic biodegradable polymers among the other known by-products—lactic acid, carboxylic acid, amine, diols, acetic acid, acetaldehyde, and Ԑ-hydroxycaproic acid. There are several physiological metabolisms and pathways to remove these degradation by-products depending on their chemical affinity, physical size, and solubility.

Renal or kidney clearance is the main route for the clearance of water-soluble by-products [55]. While the metabolised carbon dioxide by-products will be removed from the human body through the respiratory system, some degradation by-products do not directly exit the human body but instead are removed through consecutive metabolic cycles such as the Krebs cycle. Larger and partially hydrophobic or reactive breakdown by-products are taken up by the liver for detoxification, conjugation, and bile excretion [144]. If the degradation produces insoluble or particulate by-products, the immune system will be responsible for uptake and clearance through the phagocytic actions of macrophages and neutrophils, such as the by-products of graphene and carbon nanotubes (CNT) [145]. Of note, some of the degradation by-products are diluted and buffered prior to systemic clearance. Tailoring the degradation kinetics of synthetic polymers with the tissue regeneration timeline should also consider the clearance routes of degradation by-products. This consideration is crucial to ensure that the metabolic and excretory systems can efficiently eliminate by-product compounds without causing local accumulation or systemic toxicity. Moreover, understanding these pathways helps in selecting polymers whose degradation aligns with the capacity of physiological clearance.

## Conclusions

The successful application of biodegradable synthetic polymers in biomedical and TE hinges on achieving an optimal balance between scaffold degradation and host tissue regeneration. As demonstrated in this review, understanding the intrinsic healing timelines of different tissues, together with the degradation behaviours of polymers such as PLA, PVA, PCL, and PU, is essential for precise material selection and scaffold design. Each polymer presents distinct degradation kinetics, mechanical properties, and modification potentials, enabling their use across a spectrum of applications from short-term soft tissue repair to long-term load-bearing and structural regeneration. Tailoring factors such as crystallinity, porosity, chemical composition, and surface chemistry provides a versatile toolkit for aligning degradation rates with physiological needs. Ultimately, integrating materials science, degradation chemistry, and tissue biology will ensure the designed polymeric scaffolds are sufficient in maintaining structural integrity during physiological healing phases, degrade in synchrony with tissue maturation, and minimise adverse responses, thus enhancing functional restoration and long-term clinical success. While this review focuses on selected biodegradable polymers, the underlying design principles and mechanisms discussed may also be applicable to other synthetic polymeric systems. The authors therefore encourage future studies to extend these concepts to additional biodegradable polymers beyond the scope of the present review.

## Data Availability

All data are made available within the article.

## Abbreviations

TE: Tissue engineering
3D: Three-dimensional
ECM: Extracellular matrix
PLA: Poly(lactic) acid
PVA: Polyvinyl alcohol
PCL: Polycaprolactone
PU: Polyurethane
PGA: Polyglycolic acid
ROS: Reactive oxygen species
H^+^ ions: Hydrogen ions
ROP: Ring opening polymerisation
PLLA: Poly(L-lactide)
PDLA: Poly(D-lactide)
PDLLA: Poly(D, L-lactide)
H_2_O: Water
CO_2_: Carbon dioxide
CaCO_3_: Calcium carbonate
β -TCP: β-Tricalcium phosphate
PBS: Phosphate buffer saline
HBSS: Hank’s balanced salt solution
Mn: Manganese
PEG: Polyethylene glycol
dECM: Derived extracellular matrix
SBF: Simulated body fluid
PRP: Platelet-rich plasma
-OH: Hydroxyl
NaOH: Sodium hydroxide
CH_3_OH: Methanol
BCP: Bi-calcium phosphate
CNT: Carbon nanotubes
EDC: 1-Ethyl-3-(3-dimethylaminopropyl)-carbodiimide
NHS: N-Hydroxysuccinimide
Sn(Oct)_2_: Stannous octoate
PDA: Polydopamine
PEI: Polyethyleneimine
PEE: Polyethylene ether
O₂⁻: Superoxide anions
H₂O₂: Hydrogen peroxide
•OH: Hydroxyl radicals
HEMA: 2-Hydroxyethyl methacrylate
DEG: Diethylene glycol
NTA-DEG: Nitrilotriacetic acid-modified DEG
PLGA: Poly(lactic-*co*-glycolic acid)
MWCNTs: Multi-walled carbon nanotubes
NaN_3_: Sodium azide
BDNF: Brain-derived neurotrophic factor
BMP-2: Bone morphogenetic protein-2
BMP-7: Bone morphogenetic protein-7
CNS: Central nervous system
EGF: Epidermal growth factor
FGF: Fibroblast growth factor
GDNF: Glial cell line-derived neurotrophic factor
HGF: Hepatocyte growth factor
IGF-1: Insulin-like growth factor-1
MSC: Mesenchymal cells
NGF: Nerve growth factor
PDGF: Platelet-derived growth factor
PNS: Peripheral nervous system
TGF-β: Transforming growth factor-beta
VEGF: Vascular growth factor
CNT: Carbon nanotube
